# Galectin-9 prolongs the survival of septic mice by expanding tim-3-expressing natural killer T cells and PDCA-1^+^ CD11c^+^ macrophages

**DOI:** 10.1186/cc13147

**Published:** 2013-12-09

**Authors:** Takashi Kadowaki, Asahiro Morishita, Toshiro Niki, Junko Hara, Miwa Sato, Joji Tani, Hisaaki Miyoshi, Hirohito Yoneyama, Tsutomu Masaki, Toshio Hattori, Akihiro Matsukawa, Mitsuomi Hirashima

**Affiliations:** 1 Department of Immunology and Immunopathology, Kagawa University Faculty of Medicine, Kagawa, Japan; 2 Department of Gastroenterology and Neurology, Kagawa University Faculty of Medicine, Kagawa, Japan; 3 Department of Pathology and Experimental Medicine, Graduate School of Medicine, Okayama University, Okayama, Japan; 4 Department of Emerging Infectious Diseases, Graduate School of Medicine, Tohoku University, Sendai, Japan; 5 Laboratory of Biodefense Research, Faculty of Pharmaceutical Sciences at Kagawa Campus, Tokushima Bunri University, Kagawa, Japan

## Abstract

**Introduction:**

Galectin-9 ameliorates various inflammatory conditions including autoimmune diseases by regulating T cell and macrophage/dendritic cell (DC) functions. However, the effect of galectin-9 on polymicrobial sepsis has not been assessed.

**Methods:**

We induced polymicrobial sepsis by cecal ligation and puncture (CLP) in mice. The survival rate was compared between galectin-9- and PBS-treated CLP mice. An ELISA was used to compare the levels of various cytokines in the plasma and culture supernatants. Fluorescence-activated cell sorting analysis was further performed to compare the frequencies of subpopulations of spleen cells.

**Results:**

Galectin-9 exhibited a protective effect in polymicrobial sepsis as demonstrated in galetin-9 transgenic mice and therapeutic galectin-9 administration. In contrast, such effect was not observed in nude mice, indicating the involvement of T cells in galectin-9-mediated survival prolongation. Galectin-9 decreased TNFα, IL-6, IL-10 and, high mobility group box 1 (HMGB1) and increased IL-15 and IL-17 plasma and spleen levels. Galectin-9 increased the frequencies of natural killer T (NKT) cells and PDCA-1^+^ CD11c^+^ macrophages (pDC-like macrophages) but did not change the frequency of CD4 or CD8 T cells, γδT cells or conventional DC. As expected, galectin-9 decreased the frequency of Tim-3^+^ CD4 T cells, most likely Th1 and Th17 cells. Intriguingly, many spleen NK1.1^+^ NKT cells and pDC-like macrophages expressed Tim-3. Galectin-9 increased the frequency of Tim-3-expressing NK1.1^+^ NKT cells and pDC-like macrophages. Galectin-9 further increased IL-17^+^ NK1.1^+^ NKT cells.

**Conclusion:**

These data suggest that galectin-9 exerts therapeutic effects on polymicrobial sepsis, possibly by expanding NKT cells and pDC-like macrophages and by modulating the production of early and late proinflammatory cytokines.

## Introduction

Sepsis is the leading cause of death in critically ill patients, and the incidence of sepsis is increasing. The mortality rate of severe sepsis is very high, up to 70%. Two types of animal sepsis model have been established: the lipopolysaccharide(LPS)-induced inflammation, and the cecal ligation and puncture (CLP) model of microbial sepsis. LPS stimulates macrophages to release large amounts of TNFα and IL-1β that can precipitate tissue injury and lethal shock. Antagonists of TNFα and IL-1β have shown limited efficacy in clinical trials, most likely because these cytokines are early mediators in sepsis pathogenesis [1,2].

On the other hand, high mobility group box 1 (HMGB1) is thought to be a late mediator of endotoxin lethality in mice, and HMGB1 is first detectable in the circulation 8 hours after the onset of sepsis disease, subsequently increasing to plateau levels from 16 to 32 hours [3]. Administration of HMGB1-specific neutralizing antibodies beginning 24 hours after the onset of sepsis induced by CLP was shown to lead to a dose-dependent rescue of mice from lethal sepsis [4-6].

Recent studies have also shown that programmed death-1 (PD-1) expression on macrophages is critically associated with altering microbial clearance and the innate inflammatory response to sepsis in CLP mice [7]. Upregulation of PD-1 on T cells and the PD-ligand (L) 1 on monocytes in patients with septic shock has also been observed [8], and it has been shown that PD-1 levels correlate with increased mortality, nosocomial infections, and immune dysfunction in patients with septic shock [9]. Moreover, blockade of the PD-1/PD-L1 pathway improves survival in CLP mice by reversing immune dysfunction [10-12].

Galectin-9 (Gal-9) is a member of the galectin family that selectively binds to β-galactoside [13]. Gal-9 was first identified as an apoptosis-inducing factor for thymocytes [14] and an eosinophil-activating factor [15]. However, recent experiments have revealed that Gal-9 is a ligand of Tim-3 that is expressed on Th1 and Th17 cells, and that Gal-9 signaling induces death of these cells, resulting in the suppression of Th1- and Th17-related cytokine production *in vivo* and *in vitro*[16,17]. More recently, it has been shown that Gal-9 derived from surface Gal-9-expressing Th cells (ThGal-9) suppresses the development of Th17 cells and enhances the development of FoxP3^+^ regulatory T cells (Tregs) in a Tim-3-independent manner [18]. In addition to the above mechanisms, Gal-9 modulates immune responses by expanding myeloid suppressor cells [19] and plasmacytoid dendritic cell (pDC)-like macrophages [20-23]. We have also shown that Gal-9 ameliorates LPS-induced inflammation and protects mice from an LPS-induced Schwartzman reaction by suppressing the production of proinflammatory cytokines from LPS-stimulated macrophages and activating PGE2-producing neutrophils, respectively [24]. Therefore, it is of interest to examine the role of Gal-9 in a murine model of polymicrobial sepsis induced by CLP. The purpose of the present experiment is to show a beneficial effect of Gal-9 in a murine sepsis model induced by CLP and to clarify possible functions of Gal-9 in this model.

## Materials and methods

### Mice

Female wild-type (WT) Balb/c mice and Gal-9 transgenic (TG) mice were used in the present study [24]. These mice were maintained on a 12:12-hour light–dark cycle under specific pathogen-free conditions at Kagawa University. The animals were fed a standard laboratory diet and were provided water *ad libitum*. All experimental procedures were approved by the Animal Care and Use Committee of Kagawa University. The animals used in this research received humane care in accordance with international guidelines and national law.

### Induction of sepsis

CLP appears to be a reliable and clinically relevant animal model of the human septic condition because it produces an endogenous polymicrobial infection similar to clinical peritonitis and sepsis. Thus, sepsis was induced by CLP in the present experiments as described elsewhere [25]. Briefly, mice were anesthetized, and the cecum was exposed, ligated with a 3–0 silk suture and punctured once with an 18-gauge needle. The cecum was then replaced in the peritoneal cavity, and the incision was closed with surgical staples. All mice received a subcutaneous injection of 1 ml of sterile saline to avoid dehydration and were placed on a heating pad until they recovered from anesthesia. All mice were allowed free access to food and water throughout the experiments. We used stable human Gal-9 [26] to assess the effects of Gal-9 on the survival of CLP mice. The purity of Gal-9 was >95%. The endotoxin level was determined using a Limulus assay (BioWhittaker, Tokyo, Japan) and was found to be <0.008 endotoxin units. CLP mice were treated with Gal-9 intravenously or subcutaneously immediately after CLP unless otherwise specified. To determine the survival of the mice, CLP mice were monitored for 7 days after CLP. In a different set of experiments, CLP mice were anesthetized, bled and euthanized at indicated time points after CLP.

### Bacterial colony-forming unit determination

The peritoneal cavities were washed with 2 ml of sterile saline, and the lavage fluids were harvested. A 10 μl aliquot of lavage fluids was used to assess bacterial colony-forming units (CFU). Peritoneal fluids (PF, 10 μl) from each mouse were serially diluted with sterile saline. Each PF dilution was plated on tryptic soy blood agar plates and incubated overnight at 37°C, after which the number of aerobic colonies was counted. Data were expressed as CFU per 10 μl PF.

### Cell preparation

Spleens were obtained at 24 hours after CLP from mice treated with PBS or Gal-9 immediately after CLP and dispersed into single-cell suspensions. Red blood cells (RBC) were lysed with 0.1 N NH_4_Cl, and the spleen cells were washed three times with Roswell Park Memorial Institute medium (RPMI) 1640 and cultured in RPMI 1640 solution supplemented with 5% FCS, glutamine and antibiotics in a 5% CO_2_ incubator for 48 hours.

### Measurement of cytokines

Murine cytokines were quantitated using a standard sandwich ELISA according to the manufacturer’s protocol. The capture antibodies (Abs), detection Abs and the recombinant cytokines were purchased from R&D Systems or BioLegend. The ELISAs used in this study did not cross-react with other available murine cytokines.

### Flow cytometry analysis

Spleen cells were harvested from WT and Gal-9 TG CLP mice at 24 hours after CLP, and the cells (1 × 10^6^ cells/ml) were suspended in PBS supplemented with 2% FCS and 0.1% sodium azide. Cells were stained with mAbs specific for mouse CD3, CD4, CD8, GL-3, NK1.1 and Tim-3 (BioLegend, Tokyo, Japan). Stained cells were analyzed using a FACSCalibur (BD Biosciences, Tokyo, Japan). To assess intracellular IL-17 production, spleen cells were cultured for 48 hours in the presence of 1 μg/ml brefeldin A (Sigma, Tokyo, Japan) and were permeabilized overnight with permeabilization buffer (Cytofix/Cytoperm; eBioscience, San Diego, CA, USA).

### Statistics

Statistical significance was determined using the Mann–Whitney *U*-test. In the case of survival curves, the data were analyzed using the log-rank test. A *P-*value <0.05 was regarded as statistically significant. All data were expressed as mean ± standard error of the mean (SEM).

## Results and discussion

### Improved survival in CLP-induced sepsis in Gal-9 TG mice

**Figure 1:**
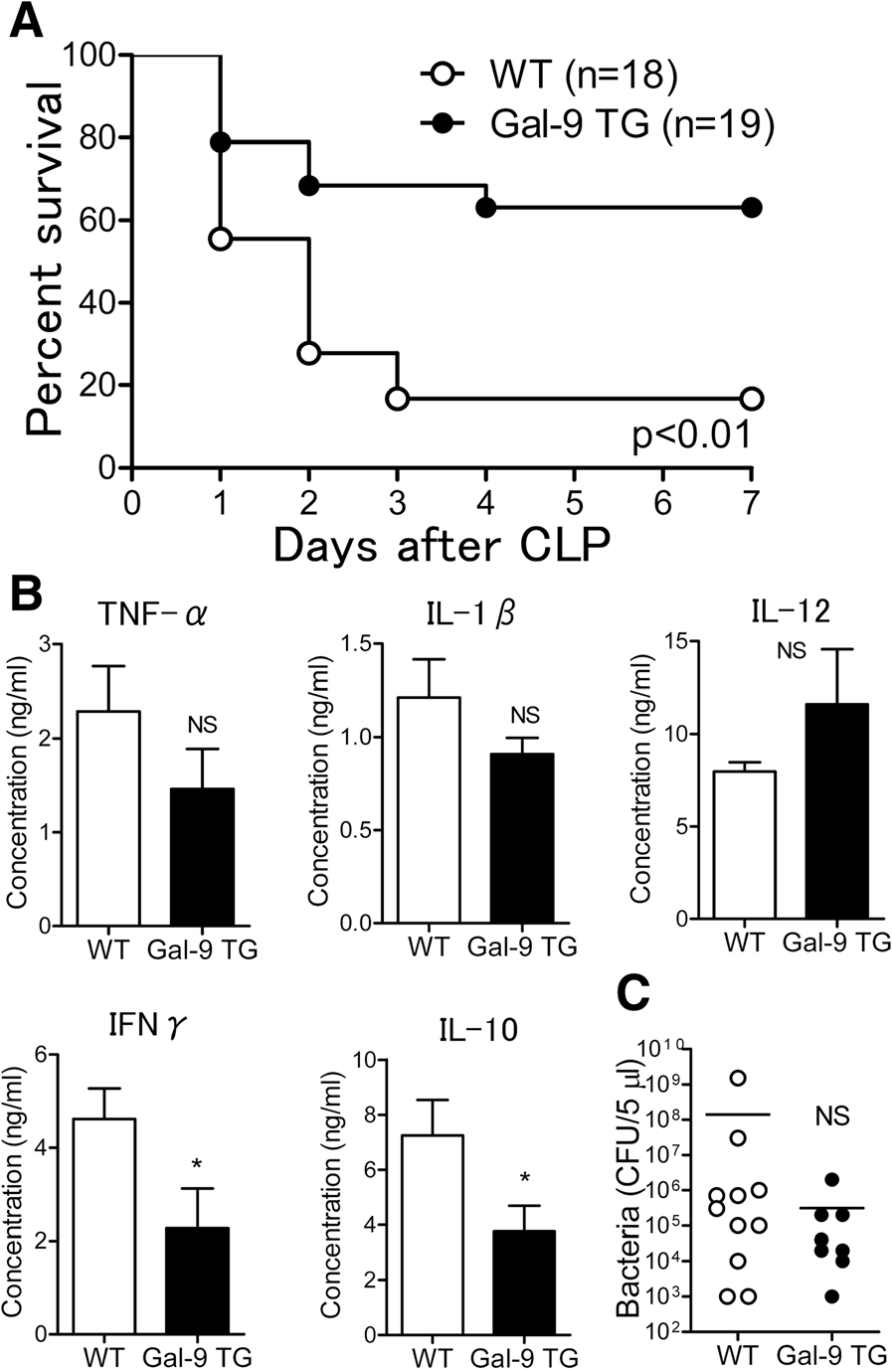
**Survival of galectin (Gal)-9 transgenic (TG) mice during polymicrobial sepsis induced by cecal ligation and puncture (CLP). (A)** Prolonged survival of Gal-9 TG mice. CLP was performed, and survival was monitored for 7 days after CLP in wild-type (WT) and Gal-9 TG CLP mice. The log-rank (Mantel-Cox) test was used for statistical analysis; *P* <0.01. **(B)** Cytokine levels in peritoneal fluids (PF) from WT (n = 18) and Gal-9 TG (n = 19) mice. At 24 hours after CLP, PF was obtained and ELISA was used for quantification of cytokine levels (^*^P <0.05; NS, not significant). **(C)** Bacterial load in WT (n = 11) and Gal-9 TG (n = 8) mice. PF dilutions were plated on tryptic soy blood agar plates and incubated overnight at 37°C. The number of aerobic colonies was counted.

We first compared the survival rate after CLP between (WT and Gal-9 TG mice. The survival rate of Gal-9 TG mice was significantly higher than that of WT mice. Of the 19 Gal-9 TG mice, 12 survived to day 7 after CLP (63.2%), whereas only 3 of 18 WT mice survived to day 7 (16.7%) (*P* <0.01). Thus, Gal-9 TG mice were resistant to the lethality induced by CLP, thereby suggesting a beneficial effect of Gal-9 administration in mice undergoing CLP (Figure 1A).

To uncover the mechanism by which Gal-9 prolongs the survival of CLP mice, we assessed the levels of pro-inflammatory cytokines such as TNF-α and IL-1β in the PF of WT and Gal-9 TG mice at 24 hours after CLP. Figure 1B shows that the levels of TNF-α and IL-1β were relatively decreased at this time point and that the level of IL-12 was relatively increased in Gal-9 TG mice compared to WT mice. However, we previously showed that the levels of TNF-α and IL-12 in PF were significantly suppressed in Gal-9 TG mice during early periods (1 to 6 hours) of LPS-induced peritoneal inflammation [24]. In contrast, the levels of IFNγ and IL-10 were significantly decreased in Gal-9 TG mice.

We further tested whether Gal-9 could decrease the bacterial load in PF at 24 hours after CLP. The bacterial load in Gal-9 TG mice tended to be lower than the bacterial load in WT mice but the difference was not statistically significant (Figure 1C). No bacterial CFU or few bacterial CFU were found at 7 days after CLP in the PF of Gal-9-treated surviving mice, as expected (data not shown).

### Delayed Gal-9 treatment prolongs the survival of CLP mice

**Figure 2:**
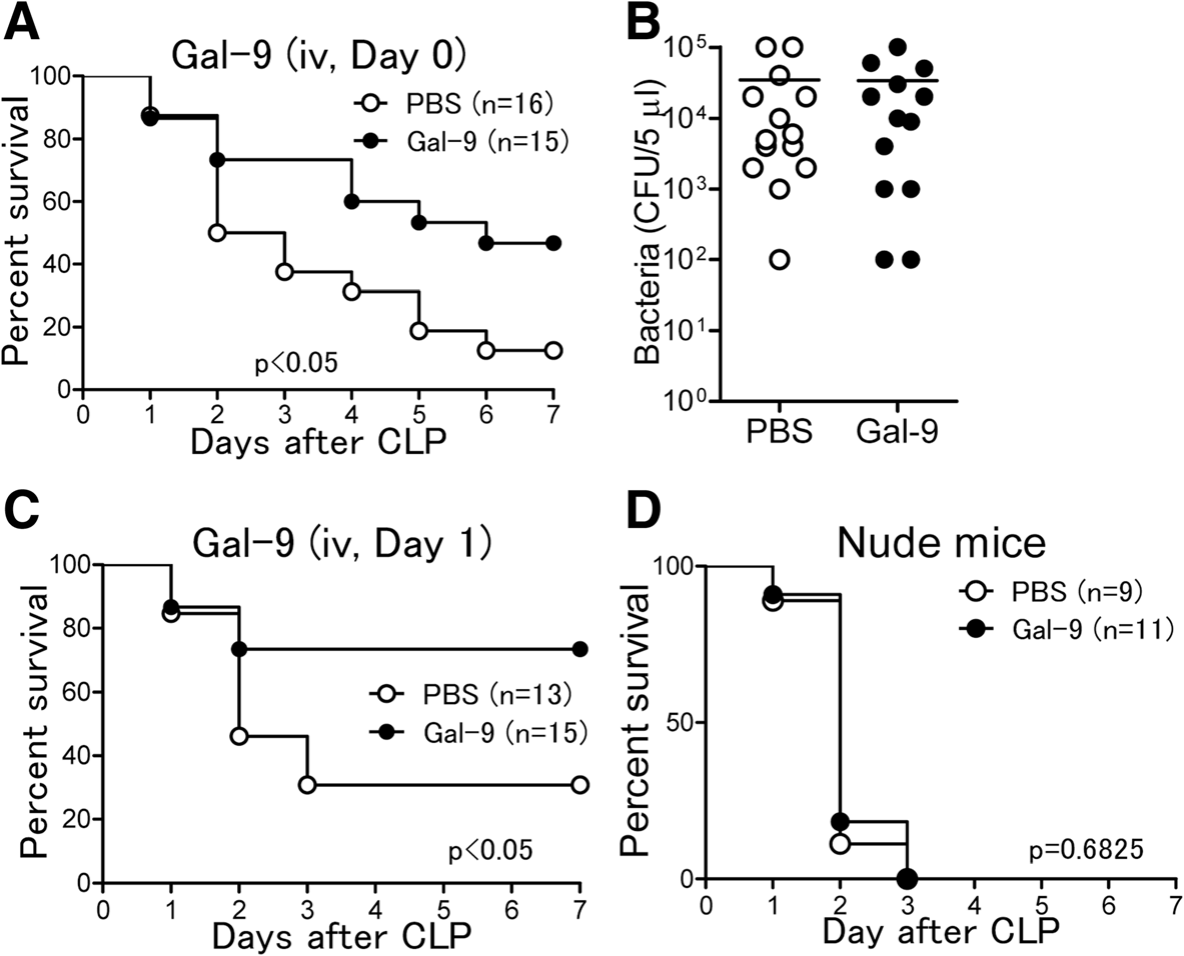
**Delayed galectin (Gal)-9 treatment prolongs the survival of cecal ligation and puncture (CLP) mice. (A)** CLP mice received a single Gal-9 injection (intravenous (i.v.), 30 μg/mouse) immediately after CLP, and survival was monitored for 7 days. The log-rank (Mantel-Cox) test was used for statistical analysis. **(B)** Bacterial load in peritoneal fluid (PF) in PBS- (n = 14) and Gal-9-treated (n = 13) mice at 24 hours after CLP. PF dilutions were plated on tryptic soy blood agar plates and incubated overnight at 37°C. The number of aerobic colonies was counted (NS, not significant). **(C)** Therapeutic effects of delayed i.v. Gal-9 injection on septic mice induced by CLP. Gal-9 was injected i.v. at 24 hours after CLP, and survival was monitored for 7 days. The log-rank (Mantel-Cox) test was used for statistical analysis; *P* <0.05. **(D)** No therapeutic effects of i.v. Gal-9 injected immediately after CLP in nude mice. The log-rank (Mantel-Cox) test was used for statistical analysis of survival.

To determine whether Gal-9 exhibits protective effects in CLP mice, we treated CLP mice with a single Gal-9 administration (intravenous (i.v.), 30 μg/mouse) immediately after CLP. As shown in Figure 2A, 7 of 16 Gal-9-treated CLP mice survived at day 7 after CLP (43.8%), whereas only 2 of 16 PBS-treated mice survived at this time point (12.5%) (*P* <0.05). We further failed to detect any significant difference in the bacterial loads of the PF between PBS- and Gal-9-treated mice at 24 hours after CLP (Figure 2B). Similar to Gal-9 TG mice, no bacterial CFU or few bacterial CFU were found at 7 days after CLP in the PF of Gal-9-treated surviving mice (data not shown). These results suggest that direct bacterial suppression by Gal-9 is not the trigger for this prolonged survival. Further studies are required to ascertain the mechanisms how Gal-9 finally decreases bacterial CFU on Day 7.

Furthermore, delayed i.v. Gal-9 administration given at 24 hours after CLP also significantly prolonged the survival of CLP mice: 11 of 15 Gal-9-treated mice survived at day 7 after CLP (73.3%), whereas only 4 of 16 PBS-treated mice survived at this time point (25%) (*P* <0.05) (Figure 2C). Moreover, we found that delayed subcutaneous Gal-9 treatment given at 24 hours after CLP similarly prolonged the survival of CLP mice: 15 of 22 Gal-9-treated mice survived at day 7 after CLP (68.2%), whereas 8 of 22 PBS-treated mice survived at this time point (40.0%) (*P* = 0.058). These results suggest that Gal-9 has therapeutic effects on microbial sepsis, probably by modulating the immune response to prolong the survival of CLP mice.

Interestingly, Gal-9 did not exhibit any therapeutic effects in nude mice: All animals that received Gal-9 administration died by 3 days after CLP similar to CLP mice that did not receive Gal-9 (Figure 2D), suggesting that T cells are critically involved in the survival enhancement mediated by Gal-9.

### Effects of Gal-9 on plasma levels of cytokines

**Figure 3:**
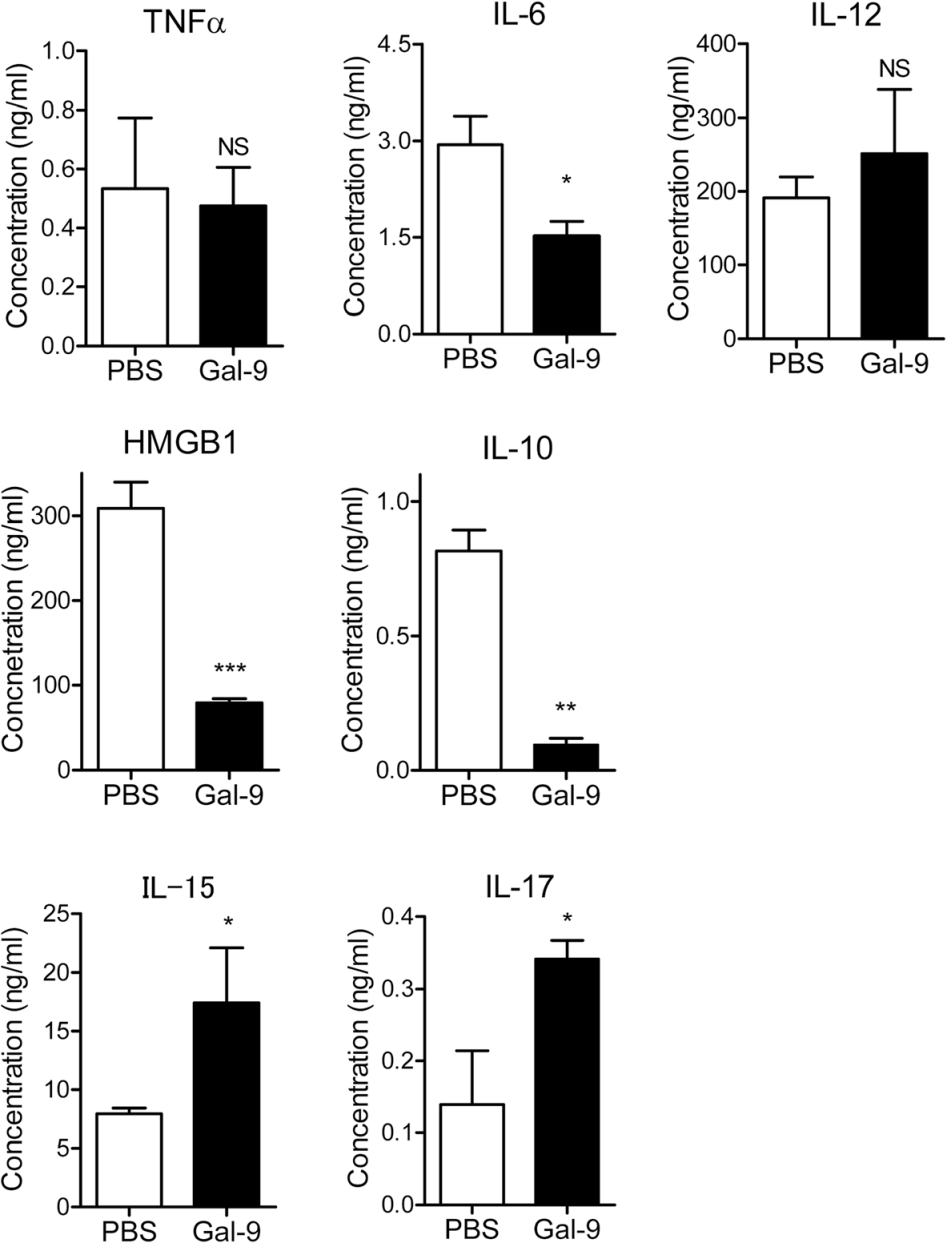
**Effects of galectin (Gal)-9 on plasma cytokine levels in cecal ligation and puncture (CLP) mice.** Plasma was prepared at 24 h after a single intravenous (i.v.) Gal-9 injection (i.v., 30 μg/mouse) immediately after CLP, and plasma cytokines were assessed by ELISA analysis; n = 8 to 12; NS, not significant; ^*^P <0.05; ^**^P <0.01; ^***^P <0.001). HMGB1, high mobility group box 1.

To determine the functions of Gal-9 in CLP mice, plasma levels of pro-inflammatory cytokines were assessed. Similar to the Gal-9 TG mice, Gal-9 failed to affect the levels of early pro-inflammatory cytokines such as TNF-α at 24 hours after CLP, although the level of IL-6 was suppressed by Gal-9 (Figure 3). In contrast, the level of HMGB1, a late inflammatory mediator [3], was markedly suppressed in Gal-9-treated mice at 24 hours after CLP (Figure 3). This result seems reasonable because delayed administration of antibodies against HMGB1 attenuates endotoxin lethality [3] and CLP-induced sepsis in rodents [4,5]. Furthermore, we found a decreased level of IL-10 and increased levels of IL-15 and IL-17 in Gal-9-treated CLP mice.

Based on the above results, we hypothesized that Gal-9 plays a critical role in not only the early but also in the late stage of sepsis, in which HMGB1 plays a critical role [27] to protect mice from lethality. This conclusion is based on our previous findings that Gal-9 administration suppresses the production of pro-inflammatory cytokines (TNF-α, IL-12 and IFNγ) during early time points (1 to 6 hours) following LPS-induced inflammation [24].

### Effects of Gal-9 on cytokine production from spleen cells

**Figure 4:**
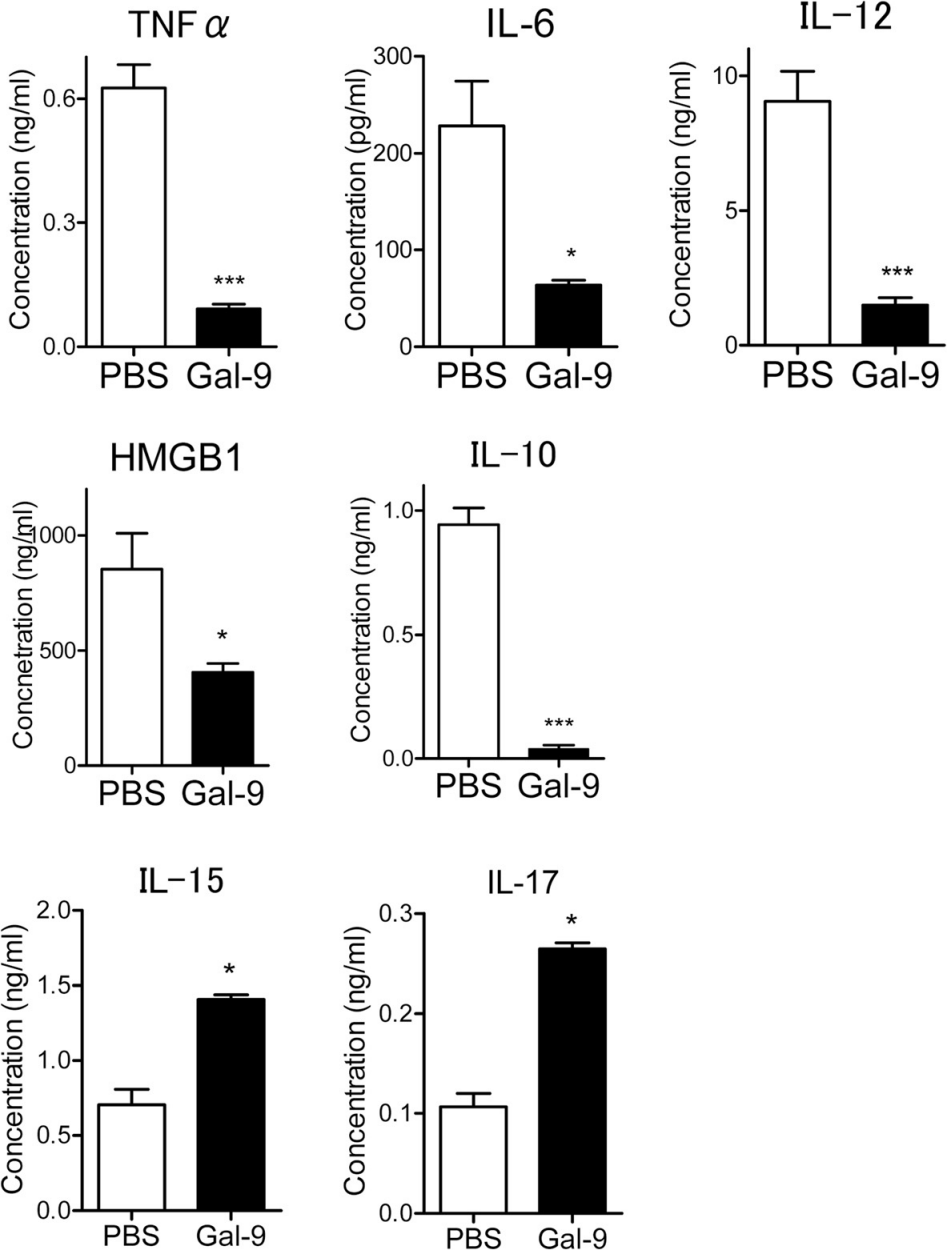
**Effects of galectin (Gal)-9 on cytokine production from spleen cells.** Spleen cells were obtained at 24 hours after cecal ligation and puncture and PBS or Gal-9 injection. These cells were then cultured without any stimulation for 48 hours, and an ELISA assay was used for quantification of cytokine levels released by the spleen cells; n = 6 to 8; NS, not significant; ^*^P <0.05; ^***^P <0.001.

The next set of experiments was performed to compare the levels of cytokine production from spleen cells from PBS- and Gal-9-treated CLP mice. Spleen cells were obtained at 24 hours after CLP and cultured without any stimulation for 48 hours. Spleen cells from Gal-9-treated CLP mice released decreased levels of early and late proinflammatory cytokines such as TNFα, IL-6, IL-12 and HMGB1 (Figure 4). The level of IL-10 was also decreased in Gal-9-treated CLP mice (Figure 4). Although IL-10 is an immunosuppressive cytokine, neutralization of IL-10 leads to improved survival of septic mice by restoring the downregulation of the IL-18 receptor on NK cells and IFN-γ production [28,29].

In contrast, an increase in IL-17 production was observed from spleen cells from Gal-9-treated CLP mice (Figure 4), which is unexpected because Gal-9 reduces IL-17-producing Th17 cells by inducing apoptosis and downregulating the differentiation of Th17 cells [17,18]. IL-17 is a proinflammatory cytokine produced during acute, delayed and autoimmune inflammation [30]. In addition to Th17 cells, other T cells such as CD8 T cells (Tc17) [31,32], natural killer T (NKT) cells [33,34] and γδT cells [35,36] could be the source of IL-17. In autoimmune diseases, it is important to reduce pro-inflammatory IL-17 levels to improve disease severity [17]. In contrast, IL-17 plays a critical role in protecting mice from sepsis-mediated lethality [37,38].

Furthermore, Gal-9 treatment resulted in an increase in IL-15 production compared to PBS-treated mice (Figure 4). IL-15 is an anti-apoptotic cytokine that prevents apoptosis by inducing apoptosis inhibitors such as Bcl-2 [39]. Inoue *et al*. [40] recently showed that IL-15 prevents apoptosis, reverses innate and adaptive immune dysfunction and improves survival in a sepsis model. Therefore, it seems reasonable that there is upregulation of IL-17 and IL-15 in Gal-9-treated CLP mice that are resistant to CLP-induced lethality.

The level of IL-10 was suppressed in Gal-9-treated CLP mice (Figure 4). Although IL-10 is an immunosuppressive cytokine, neutralization of IL-10 leads to improved survival by restoring the downregulation of the IL-18 receptor on NK cells and IFN-γ production in septic mice [28,29].

Based on the present results, it is thus suggested that it is important to increase IL-15 and IL-17 production and to decrease IL-10 and HMGB1 production simultaneously to protect mice from CLP-induced lethality. Furthermore, as we have previously proposed [41], Gal-9 should be regarded as a homeostasis-maintaining factor to keep an adequate immune response, not just as an immunosuppressive factor or immunostimulatory factor.

### Effects of Gal-9 on spleen T cells

**Figure 5:**
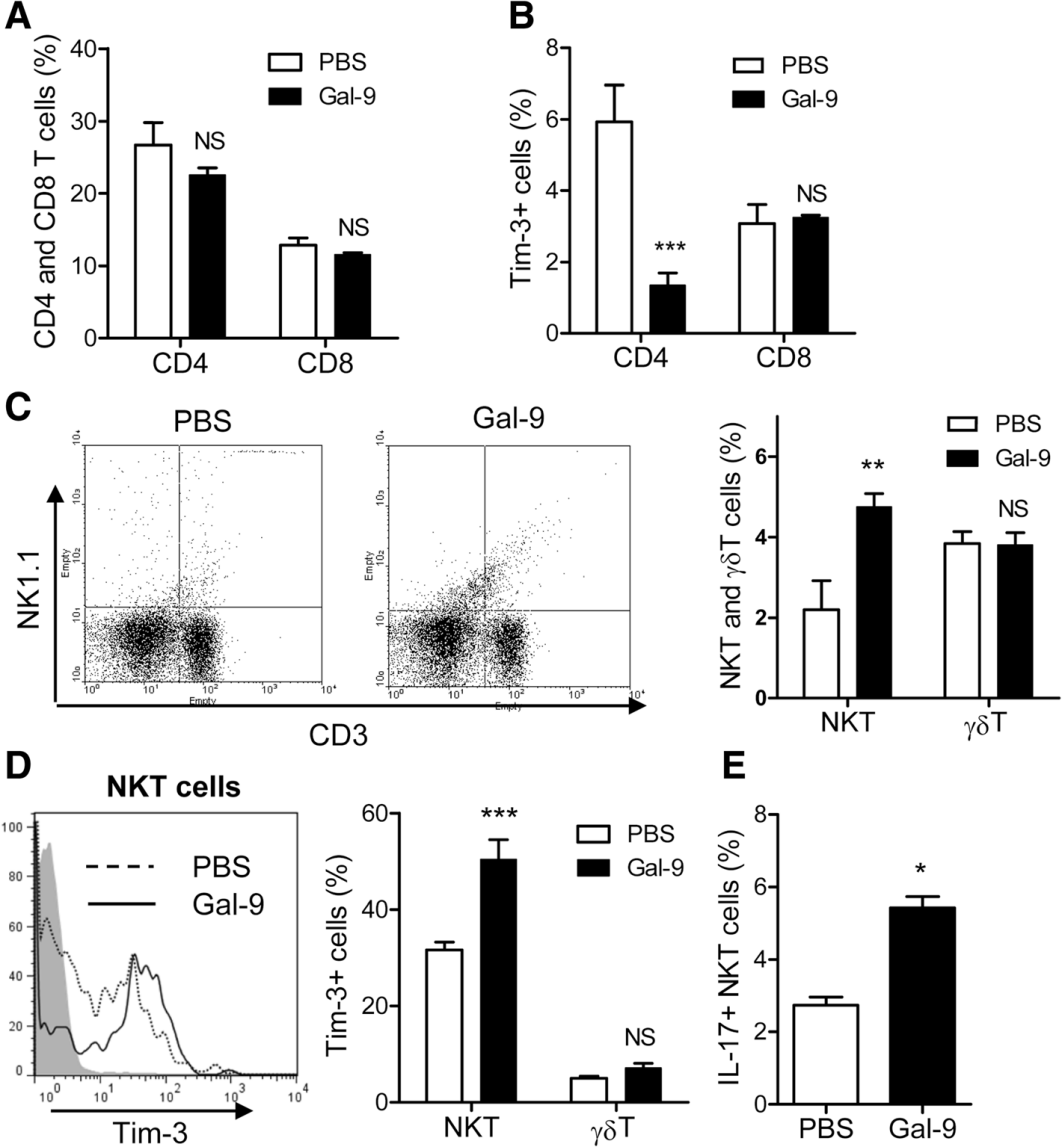
**Effects of galectin (Gal)-9 on subpopulations of spleen T cells.** Spleen cells were obtained from PBS- or Gal-9-treated cecal ligation and puncture (CLP) mice at 24 hours after a single intravenous injection. Flow cytometric analysis was performed to clarify which types of T cells are expanded by Gal-9 in the spleens of CLP mice. **(A)** The frequencies of CD4 and CD8 T cells. Spleen cells were stained with CD3, CD4, CD8 and Tim-3, and fluorescence-activated cell sorting (FACS) analysis was performed; n = 5 for each group (NS, not significant). **(B)** The frequency of Tim-3+ CD4 and CD8 T cells. Spleen cells were stained with CD3, CD4, CD8 and Tim-3, and FACS analysis was performed; n = 5 for each group (NS, not significant; ^***^P <0.001). **(C)** The frequencies of natural killer T (NKT) cells and γδT cells. Spleen cells were stained with CD3, GL-3 and NK1.1, and FACS analysis was performed; n = 5 for each group (NS, not significant; ^**^P <0.001). **(D)** Expansion of Tim-3+ NKT cells but not γδT cells. Spleen cells were stained with CD3, GL-3, NK1.1 and Tim-3, and FACS analysis was performed; n = 5 for each group (NS, not significant; ^***^P <0.001). **(E)** Gal-9 treatment resulted in an increase of intracellular IL-17+ NKT cells. Spleen cells were stained with CD3, NK1.1 and intracellular IL-17, and FACS analysis was performed; n = 5 for each group (^*^P <0.05).

As noted above, there are several types of T cells: CD4 T cells (CD3^+^ CD4^+^), CD8 T cells (CD3^+^ CD8^+^), γδT cells (CD3^+^ GL-3^+^) and NKT cells (CD3^+^ NK1.1^+^). Spleen cells were obtained 24 hours after CLP. Flow cytometric analysis was performed to clarify which types of T cells are expanded by Gal-9 in CLP mice. The frequencies of CD4 and CD8 T cells were not changed by Gal-9 (Figure 5A). We also assessed the frequency of Tim-3 expression on those T cells, because Tim-3 is a ligand of Gal-9 [16]. Approximately 6% of CD4 T cells expressed Tim-3 on their surface, and Gal-9 reduced the frequency of Tim-3^+^ CD4 T cells, likely Th1 and Th17 cells (Figure 5B), in agreement with our previous data that show that Gal-9 induces the cell death of both Th1 and Th17 cells through a Gal-9/Tim-3 interaction [16-18]. In contrast, Gal-9 weakly but not significantly increased Tim-3^+^ CD8 T cells in the spleen of Gal-9-treated mice (Figure 5B), suggesting that Gal-9 does not induce cell death of Tim-3^+^ CD8 T cells, at least under these conditions. Indeed, we have shown that Gal-9 potentiates CD8 T cell-mediated antitumor immunity via Gal-9-Tim-3 interactions between dendritic cells (DCs) and CD8 T cells [41].

Moreover, Gal-9 increased the frequency of CD3^+^ NK1.1^+^ cells, most likely NKT cells (Figure 5C). One third of NK1.1^+^ NKT cells expressed Tim-3, and Gal-9 significantly increased the frequency of Tim-3-expressing NK1.1^+^ NKT cells (Figure 5D). In contrast, Gal-9 did not increase the frequency of γδT cells (Figure 5C) or the Tim-3 expression of those cells (Figure 5D). We further found that Gal-9 treatment resulted in an increase in the frequency of intracellular IL-17^+^ NKT cells in the spleens of CLP mice (Figure 5E). These findings suggest that Gal-9 does not induce cell death of Tim-3-expressing NK1.1^+^ NKT cells, but enhances activation of those cells to produce IL-17. It is not surprising, because we had similar findings in our previous work, showing that Gal-9 promotes the activation of DCs to produce a small amount of TNF-α [42].

From the present experiments, NKT cells are proposed to be one of the main cell-types responsible for the Gal-9-induced prolongation of survival, by releasing IL-17 in CLP mice. Regarding the role of NKT cells in sepsis, there are competing opinions: one is that NKT cells are involved in harmful outcomes in sepsis as reviewed by Leung and Harris [43], and another is that those cells exhibit a protective function in sepsis [44,45]. Thus, it becomes important to explain such a discrepancy. NKT cells are broadly categorized into at least two groups: Type I NKT cells and Type II NKT cells [46]. Furthermore, immune response in sepsis consists of an initial hyper-reactive phase and a latent phase. The initial hyper-reactive phase is characterized by the large release of pro-inflammatory cytokines from macrophages et cetera, and the latent anti-inflammatory or immunosuppressed phase is characterized by hypotensive shock, inability to clear the infection, increased susceptibility to nosocomial infections, and possible multiple organ failure and death [47]. Our previous and present experiments suggest that Gal-9 decreases pro-inflammatory cytokine levels at the initial hyper-reactive phase [24]. In contrast, Gal-9 decreases HMGB1 but increases IL-10 and IL-17. Gal-9 also expands Tim-3^+^ NK1.1^+^ NKT cells and IL-17^+^ NKT cells. Therefore, we cannot exclude the possibility that Tim-3 expressing NK1.1^+^ NKT cells produce IL-17 to protect sepsis through the Gal-9/Tim-3 pathway. Thus, the hypothesis may be proposed that Gal-9 contributes to downregulate the early inflammatory response during sepsis but also to upregulate the delayed inflammatory response; further studies are required to clarified this.

### Effects of Gal-9 on spleen macrophages and DCs

**Figure 6:**
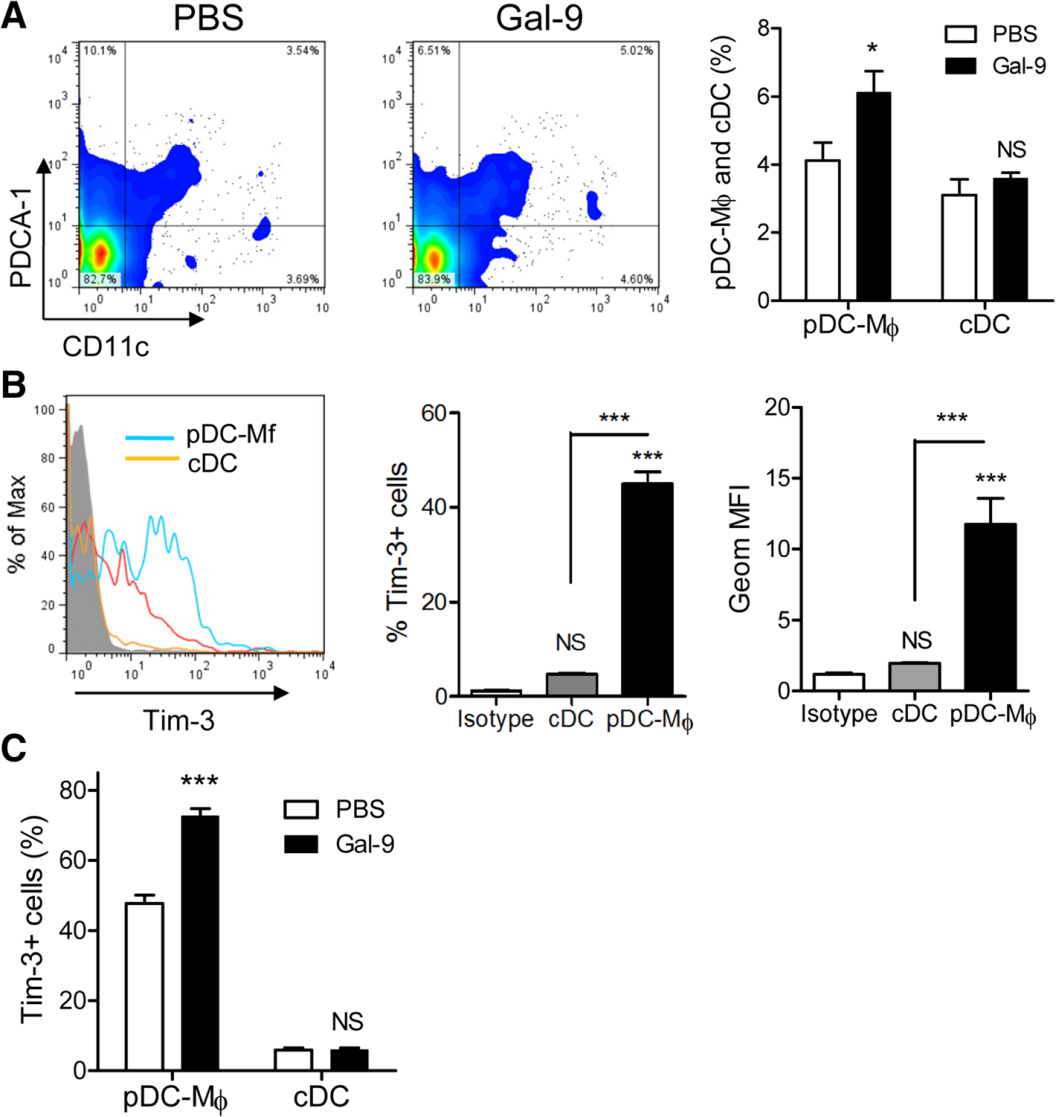
**Effects of galectin (Gal)-9 on splenic macrophages and dendritic cells (DCs).** Spleen cells were obtained from PBS- or Gal-9 treated cecal ligation and puncture (CLP) mice at 24 hours after a single intravenous injection and stained with F4/80, PDCA-1, CD11c and Tim-3. **(A)** An increased frequency of plasmacytoid (p)DC-like macrophages (F4/80+ PDCA-1+ CD11c+) but not cDCs (F4/80- PDCA-1- CD11c+) was observed; n = 5 for each group (NS, not significant; ^*^P <0.05). **(B)** High Tim-3 expression on pDC-like macrophages was observed; n = 5 for each group (NS, not significant; ^***^P <0.001). Blue line, pDC-like macrophages; orange line, cDCs; red line, PDCA-1+ CD11c- cells. **(C)** Increased Tim-3-expressing pDC-like macrophages in Gal-9-treated CLP mice; n = 5 for each group (NS, not significant; ^***^P <0.001).

Because innate immune cells such as macrophages and DCs may play a critical role in prolonging the survival of septic mice by activating/regulating T cells [7,48,49], we performed the experiments to clarify whether Gal-9 has an effect on the frequency of macrophages or DCs. Gal-9 markedly increased the frequency of plasmacytoid (p)DC-like macrophages (F4/80^+^ PDCA-1^+^ CD11c^+^), although Gal-9 failed to increase the frequency of cDCs (F4/80^-^ PDCA-1^-^ CD11c^+^) (Figure 6A).

Next, we assessed the Tim-3 expression on macrophages and DCs in the spleens of CLP mice. Intriguingly, half of the pDC-like macrophages expressed Tim-3, although cDCs did not significantly express this molecule (Figure 6B). Moreover, we compared the Tim-3 expression on splenic macrophages and DCs between the Gal-9- and PBS-treated CLP mice. Gal-9 significantly increased the frequency of Tim-3-expressing pDC-like macrophages (Figure 6C). In contrast, the frequency of Tim-3^+^ cDCs was low (Figure 6B), and Gal-9 failed to increase the frequency of Tim-3-expressing cDCs in CLP mice (Figure 6C). HMGB1 is a macrophage-derived late proinflammatory cytokine, and TLR4 and CD14 are required for the release of HMGB1 [50]. pDC-like macrophages suppress LPS-induced release of early proinflammatory cytokines from macrophages by suppressing TLR4 and CD14 expression on macrophages [21,22]. Thus, it was suggested that immunosuppressive pDC-like macrophages may mediate decreased release of early and late proinflammatory cytokines from macrophages that also, at least in part, may be involved in the prolongation of survival seen in these experiments. Of course, further studies are required to ascertain whether Tim-3^+^ NKT cells and pDC-like macrophages are indeed involved in the survival prolongation induced by Gal-9.

Furthermore, the possibility that Gal-9 blocks the PD-1/PD-L1 pathway cannot be excluded, as blocking PD-1 improves survival in CLP mice [10-12]. Therefore, further studies are required to clarify whether Gal-9 affects PD-1 and PD-L1 expression on macrophages and T cells and whether Gal-9 blocks the PD-1/PD-L1 pathway.

## Conclusions

Gal-9 exhibits therapeutic effects on CLP-induced polymicrobial sepsis, potentially by expanding NKT cells and pDC-like macrophages, and by modulating the production of early and late pro-inflammatory cytokines.

## Key messages

• Delayed Gal-9 treatment prolongs the survival of polymicrobial sepsis in mice induced by CLP.

• Gal-9 decreases TNFα, IL-6, IL-10 and HMGB1 levels but increases IL-15 and IL-17 levels.

• Gal-9 expands NKT cells and pDC-like macrophages, although it decreases the frequency of Tim-3^+^ CD4 T cells.

• Many NKT cells and pDC-like macrophages express Tim-3, and Gal-9 increases the frequency of Tim-3^+^ NKT cell and pDC-like macrophages.

## Abbreviations

CFU: Colony-forming unit; CLP: Cecal ligation and puncture; DC: Dendritic cell; ELISA: Enzyme-linked immunosorbent assay; FACS: Fluorescence-activated cell sorting; FCS: Fetal calf serum; Gal-9: Galectin-9; HMGB1: High mobility group box 1; IFN: Interferon; IL: Interleukin; i.v.: Intravenous; L: Ligand; LPS: Lipopolysaccharide; NKT: Natural killer T; PBS: Phosphate-buffered saline; PD-1: Programmed death-1; pDC: Plasmacytoid dendritic cell; PF: Peritoneal fluids; RBC: Red blood cells; RPMI: Roswell Park Memorial Institute medium; SEM: Standard error of the mean; TG: Transgenic; ThGal-9: Surface Gal-9-expressing Th cells; Tim-3: T cell immunoglobulin motif 3; TNF: Tumor necrosis factor; Tregs: Regulatory T cells; WT: Wild-type.

## Competing interests

Drs Niki and Hirashima are board members of GalPharma Co., Ltd. Although there are patents and products related to this manuscript in development, this does not alter our adherence to all of the Journal’s policies on sharing data and materials as detailed in the guide for authors. The patent for stable-form Gal-9 is issued in Japan (4792390), the USA (8,268,324), EPC (1736541), Canada (2,561,696) and Korea (10–1222281) and is applied for in China (200580010446.1). The other authors declare that they do not have any competing interests.

## Authors’ contributions

AM, MH, TM and TH designed the experiments and helped to draft the manuscript. TK and AM carried out the FACS analysis and ELISA for spleen cell culture supernatants and drafted the manuscript. JH and MS performed the *in vivo* survival test, ELISA for plasma cytokine levels and bacterial colony formation experiments using peritoneal fluids. JT, HM and HY carried out the ELISA of culture supernatants of spleen cells. TN prepared recombinant galectin-9 for the present study and helped with the FACS analysis. All authors read and approved the final manuscript.

## Authors’ information

Akihiro Matsukawa and Mitsuomi Hirashima are co-senior authors.
